# Gender-related influences on adherence to advice and treatment-seeking guidance for infants and young children post-hospital discharge in Bangladesh

**DOI:** 10.1186/s12939-021-01404-7

**Published:** 2021-02-24

**Authors:** Md. Fakhar Uddin, Sassy Molyneux, Kui Muraya, Md. Alamgir Hossain, Md. Aminul Islam, Abu Sadat Mohammad Sayeem Bin Shahid, Scholastica M. Zakayo, Rita Wanjuki Njeru, Julie Jemutai, James A. Berkley, Judd L. Walson, Tahmeed Ahmed, Haribondhu Sarma, Mohammod Jobayer Chisti

**Affiliations:** 1 Nutrition and Clinical Services Division, icddr,b, GPO Box 128, Dhaka, 1000 Bangladesh; 2 The Childhood Acute Illness and Nutrition (CHAIN) Network, Nairobi, Kenya; 3 KEMRI-Wellcome Trust Research Programme, P.O. Box 230-80108, Kilifi, Kenya; 4 Centre for Tropical Medicine and Global Health, University of Oxford, Old Road Campus, Headington, Oxford, OX3 7BN UK; 5 KEMRI-Wellcome Trust Research Programme, P.O. Box 43640-00100, Nairobi, Kenya; 6 Department of Global Health, Medicine, Paediatrics and Epidemiology, University of Washington, Seattle, USA; 7 Research School of Population Health, Australian National University, Acton, ACT, Canberra, 2601 Australia

**Keywords:** Gender relations, Treatment-seeking, Undernutrition, Children, Hospitalization, Bangladesh

## Abstract

**Background:**

Post-hospital discharge mortality risk is high among young children in many low and middle-income countries (LMICs). The available literature suggests that child, caregiver and health care provider gender all play important roles in post-discharge adherence to medical advice, treatment-seeking and recovery for ill children in LMICs, including those with undernutrition.

**Methods:**

A qualitative study was embedded within a larger multi-country multi-disciplinary observational cohort study involving children aged less than 2 years conducted by the Childhood Acute Illness and Nutrition (CHAIN) Network. Primary data were collected from family members of 22 purposively selected cohort children. Family members were interviewed several times in their homes over the 6 months following hospital discharge (total *n* = 78 visits to homes). These in-depth interviews were complemented by semi-structured individual interviews with 6 community representatives, 11 community health workers and 12 facility-based health workers, and three group discussions with a total of 24 community representatives. Data were analysed using NVivo11 software, using both narrative and thematic approaches.

**Results:**

We identified gender-related influences at health service/system and household/community levels. These influences interplayed to family members’ adherence to medical advice and treatment-seeking after hospital discharge, with potentially important implications for children’s recovery. Health service/system level influences included: fewer female medical practitioners in healthcare facilities, which influenced mothers’ interest and ability to consult them promptly for their child’s illnesses; gender-related challenges for community health workers in supporting mothers with counselling and advice; and male caregivers’ being largely absent from the paediatric wards where information sessions to support post-discharge care are offered. Gendered household/community level influences included: women’s role as primary caretakers for children and available levels of support; male family members having a dominant role in decision-making related to food and treatment-seeking behaviour; and greater reluctance among parents to invest money and time in the treatment of female children, as compared to male children.

**Conclusions:**

A complex web of gender related influences at health systems/services and household/community levels have important implications for young children’s recovery post-discharge. Immediate interventions with potential for positive impact include awareness-raising among all stakeholders – including male family members - on how gender influences child health and recovery, and how to reduce adverse consequences of gender-based discrimination. Specific interventions could include communication interventions in facilities and homes, and changes in routine practices such as who is present in facility interactions. To maximise and sustain the impact of immediate actions and interventions, the structural drivers of women’s position in society and gender inequity must also be tackled. This requires interventions to ensure equal equitable opportunities for men and women in all aspects of life, including access to education and income generation activities. Given patriarchal norms locally and globally, men will likely need special targeting and support in achieving these objectives.

## Background

Post-hospital discharge mortality risk is high among children in many low and middle-income countries (LMICs) [1–4], sometimes exceeding in-hospital mortality rates [5, 6]. Undernourished infants and young children with acute illness have a particularly high risk of death during hospital admission and after discharge, despite adherence to current WHO guidance [7]. There is a clear need for a better understanding of modifiable risk factors for mortality among acutely ill young children after hospital discharge to identify targeted interventions to reduce the mortality in resource-poor settings [8]. Mechanisms of interest for future targeting of interventions include clinical, nutritional, health system, environmental, socio-cultural and economic dimensions [8].

The available literature suggests that gender plays an important role in post-discharge adherence to medical advice, treatment-seeking and in the recovery for undernourished children in LMICs [6, 9]. *Gender* refers to the socially constructed roles, behaviours, activities, and attributes that a given society considers appropriate for men and women and people of other genders [10, 11]. Gender differs from ‘sex’, which refers to the biological and physiological characteristics that distinguish males from females [10]. Gender is known to affect how men, women and people of other genders live, work and relate to one another in all walks of life, including in relation to healthcare and the health system [11]. In healthcare and health systems, gendered power relations can influence employment patterns, impacting on the gender balance across different types of healthcare providers [12]. Gender norms and the reported sex of the provider can in turn influence interpersonal dynamics between parents and providers, which shapes parents’ treatment-related decision-making and actions. In homes and communities, gender can influence vulnerability to ill-health, household decision-making for illness, health-seeking behaviour, and utilization of health services [11]. Gendered power relations can therefore affect when a child is admitted to hospital, who brings the child to hospital and stays with him/her during hospitalisation, and adherence to advice post-discharge [13, 14].

In order to improve post-discharge outcomes for children in LMICs, it is essential to design health interventions that are targeted towards positively impacting children at risk of severe illness and mortality. Interventions need to be effective and equitable, and to be informed by contextually relevant gender analyses [15]. Previous studies conducted in Pakistan and Bangladesh have suggested that the higher numbers of male medical practitioners, particularly in rural facilities, may influence treatment-seeking patterns [16, 17]. In Ethiopia, females (and their husbands) expressed discomfort at seeking care for themselves or their children from male practitioners due to social norms around interactions between sexes and appropriate discourse with members of the opposite gender [18]. In contrast to medical providers, there are more female than male community health workers (CHWs) in many countries [19–21]. CHWs, particularly females, have been shown to play an important role in supporting households, in identifying and responding to child illnesses, and in negotiating with others in the household for advisory and financial support [19, 20, 22].

At household and community levels, important gender related influences include the role of women as the main carers of children, and – especially for young mothers - having limited access to household resources and low decision-making power [23, 24]. Furthermore, there are differences in access to funds and decision-making processes between rural and urban areas [13, 23–25]. Although dynamics are now changing as a result of more women earning their own income, particularly in urban areas, and in having some control over that income in caring for their children, gender discrepancies remain in many LMICs [15, 24–28]. Another important influence at both household and community levels in South Asian contexts is a preference for male children, which in turn results in preferential medical treatment of boys over girls [29–31]. Specifically, parents are more willing to ‘go the extra mile’ for boy children compared to girls, including seeking care from more distant or expensive facilities [32]. In these settings, higher mortality rates among girls than boys – where observed - have been attributed to longer treatment delays for girls, lower rates of hospitalization, fewer vaccinations and less nutritious diets [29–32]. In this paper, we explore gender related influences on the post-discharge experience for acutely ill young children admitted to two hospitals of International Centre for Diarrhoeal Disease Research, Bangladesh (icddr,b) in Bangladesh.

Bangladesh is a patriarchal, majority Muslim society [33]. Gender inequities are reflected in females having a lower life expectancy at birth compared to men, a disproportionate distribution of food and health care being allocated to male over female children, and higher neonatal, infant and child mortality rates among boys than girls [33]. Female literacy levels remain among the lowest in Asia and maternal mortality rates amongst the highest.

There have been numerous development initiatives designed to tackle women’s situation and strengthen gender equity in Bangladesh, with some positive outcomes. Thus a growing proportion of women are being educated, earning an income and saving money in micro-credit schemes. Indeed, women’s employment has been documented as an important contribution to the economic and social wellbeing of poor households, particularly those without a male breadwinner [34]. However, patriarchal, religious and cultural norms that support secluding women and that reinforce social standards of female modesty have undermined many initiatives [28]. For example for married men, women working can be seen as undermining his, and his families’ honour, which can lead to both men and women being stigmatised. Where women are able to earn income, they are paid less than men for equivalent work [28], and there are particular difficulties, for example for poor Muslim women in accessing even informal sector employment [35]. On marriage, a woman’s family is paid dowry and she moves into her husband’s home [36]. In the case of a divorce, many women return to their paternal home, where they, together with any children accompanying them, may be seen as a financial burden and social embarrassment [36]. Dowry may not be paid or may need refunding. Such norms continue to limit women’s social and economic autonomy from men and reinforce their subordinate position in society. These norms have important implications for decision-making and treatment-seeking for young children as we will go on to show in this paper.

## Study sites and methods

### The childhood acute illness and nutrition (CHAIN) network

This study was conducted as part of a large multi-country observational prospective cohort study undertaken by the CHAIN Network (www.chainnetwork.org). CHAIN is a multi-disciplinary research network aimed at understanding the mechanisms contributing to high child mortality in hospital and after discharge in LMICs, in order to identify children at risk and interventions to improve their survival [8]. CHAIN is leading a cohort study at nine hospital sites in Africa and South Asia, recruiting more than 3000 acutely-ill children at admission to hospital and following them up post-discharge [8]. Scheduled follow up visits are conducted at days 45, 90, and 180 after hospital discharge [8]. Children are enrolled in three strata based on anthropometric status since this is a strong marker of survival risk that encompasses both biological and social risks: severely wasted or kwashiorkor (oedematous malnutrition) (SWK)- the group we consider most vulnerable as is close to the WHO definition of ‘severe acute malnutrition’ (SAM), moderately wasted (MW) which is close to ‘moderate acute malnutrition’ (MAM) and not wasted (NW) – absence of acute malnutrition. As part of the broader goal of the CHAIN Network, a qualitative social science sub-study was undertaken in four of the nine sites (two sites in both Kenya and Bangladesh). This paper focuses on the qualitative data from the Bangladesh sites; one rural and one urban.

### The Bangladesh CHAIN sites

The two CHAIN study hospitals in Bangladesh were Dhaka (urban) and Matlab (rural) hospitals of icddr,b [8]. The Dhaka hospital is located in the capital city and the Matlab hospital about 55 km southeast of Dhaka, in a rural sub-district of Chandpur District. As described elsewhere, these hospitals provide a full range of free treatment and services for patients admitted with diarrheal disease with or without complications or associated problems or with isolated respiratory illnesses [37, 38].

Most patients admitted to these hospitals are from low socio-economic backgrounds. In Dhaka city, many of the hospital users live in one of the city’s informal settlements (sometimes referred to as ‘slums’), where 37.4% of Dhaka city dwellers live and where environments are very crowded, with poor sanitation and hygiene practices [39]. Overall, in Dhaka city, 95% people are Muslim and 5% non-Muslim (mostly Hindus) [40]. When compared to non-slum urban areas, slum-dwelling children have been reported to be more undernourished, have lower immunisation rates, and have higher rates of measles, diarrheal illness and severe dehydration [41]. Children’s mothers are more likely than rural mothers to work outside of the home earning an income [41].

The majority of children admitted to Matlab hospital of icddr,b were from the Matlab sub-district. Matlab sub-district is a rural location with no major towns or cities besides Matlab bazaar, with a population of approximately 500,000 people as of 2020; 88% of the population are Muslim, and 12% non-Muslim (mostly Hindus) [42, 43]. There is limited inter-village trade and commerce. The predominant occupations are subsistence farming and fishing [44].

Table 1 provides some key relevant socio demographic details for children enrolled in CHAIN and their family members. Almost all mothers for whom there is data reported that they were married (monogamous) (*n* = 598); while 5 reported being in a polygamous relationship and 3 that they were separated/divorced.

**Table 1:** Socio demographic details for CHAIN children and their family members

Bangladesh summary statistics (SWK, MW and NW)			
	Dhaka (***N*** = 394)	Matlab (***N*** = 314)	Total (***N*** = 708)
Biological mother	378 (95.9%)	312 (99.4%)	690 (97.5%)
Caregiver age group			
< 18	29 (7.4%)	2 (0.6%)	31 (4.4%)
18–50	361 (91.6%)	310 (98.7%)	671 (94.8%)
> 50	4 (1.0%)	2 (0.6%)	6 (0.8%)
Caregiver’s education level			
None	142 (36.0%)	50 (15.9%)	192 (27.1%)
Primary	185 (47.0%)	162 (51.6%)	347 (49.0%)
Secondary	39 (9.9%)	78 (24.8%)	117 (16.5%)
Tertiary	28 (7.1%)	24 (7.6%)	52 (7.3%)
Caregiver able to read			
No	125 (31.7%)	44 (14.0%)	169 (23.9%)
Yes	269 (68.3%)	270 (86.0%)	539 (76.1%)
Mother earns an income	319 (81.0%)	302 (96.2%)	621 (87.7%)
Mother sick	141 (35.8%)	136 (43.3%)	277 (39.1%)
Food insecurity			
Low	295 (74.9%)	311 (99.0%)	606 (85.6%)
Medium	65 (16.5%)	3 (1.0%)	68 (9.6%)
No prior treatment	30 (7.6%)	39 (12.4%)	69 (9.7%)
Prior treatment seeking			
Government facility	49 (12.4%)	43 (13.7%)	92 (13.0%)
Private facility	118 (29.9%)	47 (15.0%)	165 (23.3%)
Pharmacy/Shop	214 (54.3%)	188 (59.9%)	402 (56.8%)
Traditional	24 (6.1%)	2 (0.6%)	26 (3.7%)
Travel to this admission			
Travel means - Public	100 (25.4%)	7 (2.2%)	107 (15.1%)
Travel means - Private	9 (2.3%)	2 (0.6%)	11 (1.6%)
Travel means - Ambulance	4 (1.0%)	0 (0%)	4 (0.6%)
Travel means - Non-vehicle	351 (89.1%)	311 (99.0%)	662 (93.5%)
Travel time			
< 1 h	116 (29.4%)	85 (27.1%)	201 (28.4%)
1 h to < 2 h	181 (45.9%)	116 (36.9%)	297 (41.9%)
2 h to 4 h	96 (24.4%)	97 (30.9%)	193 (27.3%)
> 4 h	1 (0.3%)	1 (0.3%)	2 (0.3%)

### The qualitative sub-study in CHAIN

#### Data collection

This qualitative study involved family members of admitted children, community representatives and diverse health workers. We (MFU and ST) interviewed family members of 22 children purposively selected from the Bangladesh CHAIN cohort. Children and their respective families were selected during the child’s admission to maximise diversity of experience based on: nutritional status (10 SWK, 7 MW and 5 NW); household socio-economic status; geographical location (10 living in Dhaka slums and 12 living in rural villages); and any recent exposure to any major socially disruptive event such as death of a caregiver. This sampling approach was intended to ensure diverse representation of different types of families and experiences in relation to the study objective and questions. Gender of child emerged an important consideration in this study; which then prompted us to undertake additional gender analysis of the data. As such, sex of the children was not initially considered as a selection criterion; rather we aimed to ensure that we had children of both sexes in our selected sample. Thirteen of the selected children were girls and nine were boys. Nobody refused to participate in our study.

We visited consenting family members in their homes between two and four times post-hospital discharge (within the 1st week, day 45, day 90 & day 180). In homes we conducted a total of 78 in-depth interviews, primarily with the children’s main carers. Three children died and another three migrated during the follow up period, and we were therefore not able to undertake the full range of interviews with these families. In-depth interviews during the home visits covered a broad range of topics including: child health and nutrition; the child’s illness trajectory and related treatment-seeking and decision-making; experiences with the admitting hospital and the health system more broadly; as well as challenges faced during the child’s illness episode and coping strategies. Non-participant observations were also conducted at the re-admitting hospital and at the household level to give a sense of living conditions and family dynamics, community relations, as well as experiences and interactions at the hospital. Household interview data were supplemented by interviews with community representatives (local gatekeepers and stakeholders e.g. political or religious leaders, school teachers) (*n* = 6), and community health workers (*n* = 11) purposively selected from the respective villages of the selected children. Interviews were also conducted with facility health workers (*n* = 12) at the hospitals where children were admitted. The main two interviewers were a male anthropology graduate and a female sociology graduate who were experienced in conducting interviews in the local dialects. Only the interviewers and participants were present during interviews. Interviews were based on semi-structured interview guides that were amended over time. Data collection continued until we reached a point of data saturation. The average duration of an interview was 48 min. All interviews were audio-recorded and arranged at times agreed in advance with interviewees.

#### Data analysis

Interviews were transcribed verbatim and observation notes written up into detailed notes immediately after the fieldwork. Transcriptions and observation notes were reviewed to identify issues for follow up in later interviews.

We combined a narrative [45] and thematic coding approach [46] in the analysis of household data. The narrative approach involved the construction of a detailed summary for each child, drawing on all available data collected for that household. We used household stories to examine families’ overall pathways through care, and to look for patterns of similarity and difference across households, facilitated by the construction of charts (see Zakayo et al. for a more detailed explanation) [47]. For the thematic analysis, all transcriptions were coded by the research team who also conducted the interviews in NVivo11 software using a coding framework developed based on our initial study objectives and preliminary emerging themes of interest. To support the trustworthiness of the coding process, at least two people coded each transcript, compared results and resolved any discrepancies. Coded data were condensed into charts to support identification of patterns by sex of children, household structure and headship, and rural and urban context. Thus thematic coding supplemented and enriched the narrative analysis. A similar thematic analysis approach was used for the community representatives and health provider data.

For this paper, we carefully examined the household stories and charts to explore any gendered power relations at household/community and health services/system levels. To support our analysis, we drew on Morgan et al.’s (2016) gender framework (Table 2) to consider what we were learning at both levels in terms of who has what (access to resources), who does what (the division of labour and everyday practices), how values are defined (social norms, ideologies, beliefs and perceptions) and who decides (rules and decision-making, both formal and informal) [11]. This gender framework was used to examine our data for key aspects of gender power relations [11].

**Table 2:** Gender as a power relation and driver of inequality

What constitutes gendered power relations	
Who has what?	Access to resources (education, information, skills, income, employment, services, benefits, time, space, social capital etc.)
Who does what?	Division of labour within and beyond the household and everyday practices
How are values defined?	Social norms, ideologies, beliefs and perceptions
Who decides?	Rules and decision-making (both formal and informal)
How power is negotiated and changed	
Individual/People	Critical consciousness, acknowledgement/lack of acknowledgment, agency/apathy, interests, historical and lived experiences, resistance or violence
Structural/Environment	Legal and policy status, institutionalisation within planning and programs, funding, accountability mechanisms

### Ethical approval

CHAIN protocols were approved for science and ethics in all participating countries. In Bangladesh, the study was approved by the Research Review Committee and the Ethical Review Committee of icddr,b. Written informed consent was sought from participants for all in-depth interviews, observations and recordings.

## Results

Following an overview of interviewee characteristics and overall treatment-seeking patterns, we bring together our findings on gender-related influences at health-system/service and household/community levels in turn. As we will show, these gender influences are inter-related and had important implications for post-discharge adherence to advice and treatment-seeking behaviour.

### Interviewee characteristics and overall treatment-seeking patterns

The main characteristics of the 22 families involved in this study are summarized in Table 3. Of the 10 urban children, 5 were SWK, 3 were MW, and 2 were NW. Across both sites, children were admitted into hospital and enrolled into the CHAIN cohort aged 3 to 17 months, with all reported by parents as having been sick on and off since birth. Eight of the 10 children had experienced a disruption in the family within the last 2 months from the time of enrolment in this study including recent migration, separation or income earning loss among parents, maternal illness or a change of caregiver.

**Table 3:** Participant and Households Characteristics

ID	Children characteristics						Mothers characteristics					Households characteristics						
	Nutritional status	Age (months)	Sick since birth?	Other illness at admission	Sex	Caregiver	Age (years)	# of kids	Marital status	Education	Employment status	Family structure	Size	Social disruption before admission	Income source	Decision maker	Distance to icdd,b hospital (km)	Transportation type (travel to hospital)
**Urban participants and households**																		
HH52	SWK	14	Yes	Diarrhoea, fever	Girl	Mother	20	3	Married	Illiterate	Housewife	Nuclear	5	Household change	Carpenter (father)	Father	12 km	Bus, CNG (three wheelers)
HH53	SWK	6	Yes	Diarrhoea	Boy	Mother	20	1	Divorced	Primary	None	Extended	8	Household change	Garment worker (uncle & aunt)	Grandmother	5 km	Bus, CNG and Rickshaw (three wheelers)
HH54	NW	15	No	N/A	Boy	Mother	20	1	Married	Illiterate	Housewife	Nuclear	3	No	Rickshaw Puller (father)	Father	7 km	Bus & CNG
HH55	SWK	4	Yes	Diarrhoea	Girl	Maid servant	19	1	Married	Primary	Garment worker	Nuclear	4	Caregiver changed	Garment worker (father & mother)	Father	3 km	Rickshaw
HH51	MW	8	Yes	Diarrhoea, fever	Boy	Mother	17	1	Married	Primary	Housewife	Nuclear	3	Recent migration, wage loss	Bakery shop worker (father)	Father	15 km	Bus & CNG
HH59	MW	4	Yes	Diarrhoea,fever	Girl	Mother	17	1	Married	Primary	Housewife	Extended	5	Recent migration	Garment worker (father)	Father	12 km	Bus & CNG
HH60	SWK	8	Yes	Diarrhoea, pneumonia	Boy	Mother in low	35	3	Married	Illiterate	Small business	Nuclear	5	Mothers’ sickness	Car driver (father), restaurant business (mother)	Father	7 km	CNG & Bus
HH61	NW	11	No	N/A	Girl	Mother	27	3	Married	Primary	Housewife	Nuclear	5	No	Small business (father)	Father	7 km	CNG & Rickshaw
HH62	MW	4	Yes	Diarrhoea,pneumonia	Boy	Mother	35	3	Married	Illiterate	Housewife	Nuclear	5	Mothers’ sickness,caregiver change	Small business (father)	Father	13 km	Bus & CNG
HH63	SWK	7	Yes	Diarrhoea,pneumonia	Girl	Aunt	19	1	Divorced	Secondary	–	Extended	4	Wage loss	Garment worker (uncle); workshop labor (father)	Grandmother	11 km	Bus & CNG
**Rural participants and households**																		
HH04	SWK	10	Yes	Diarrhoea, fever	Girl	Mother	20	3	Married	Secondary	Housewife	Extended	9	Mother’s sickness	Day labour (father)	Grandfather	20–25 km	CNG, Rickshaw
HH06	NW	17	No	N/A	Girl	Mother	21	1	Married	Secondary	Housewife	Nuclear	3	Parents sickness	Car driver (father)	Father	18 km	Rickshaw, CNG
HH07	SWK	5	Yes	Diarrhoea	Boy	Mother	26	2	Married	Secondary	Housewife	Extended	10	Mother’s sickness	Work in abroad (father)	Grandfather	28–30 km	Rickshaw, CNG
HH09	SWK	3	Yes	Diarrhoea	Boy	Mother	20	1	Married	Primary	Housewife	Extended	4	No	Work in abroad (father)	Grandfather	15 km	CNG and Rickshaw
HH11	NW	6	No	N/A	Girl	Mother	27	3	Married	Primary	Housewife	Nuclear	5	No	Farmer (father)	Father	8 km	Rickshaw and CNG
HH12	SWK	9	Yes	Diarrhoea	Girl	Mother	23	2	Married	Secondary	Housewife	Nuclear	5	No	Clerk (father)	Father	25 km	Rickshaw and CNG
HH13	NW	8	No	N/A	Girl	Mother	22	2	Married	Secondary	Housewife	Extended	5	Parents sickness	Shop keeper (father)	Grandfather	30 km	Rickshaw and CNG
Hh10	SWK	14	Yes	Diarrhoea, fever	Girl	Mother	35	3	Married	Secondary	Housewife	Nuclear	5	N	Night guard (father)	Father	15 km	Rickshaw and CNG
HH01	MW	11	Yes	Diarrhoea, fever	Boy	Mother	23	3	Married	Secondary	Housewife	Extended	9	Mother’s sickness	Work in urban as tailor (father)	Grandmother	22 km	Rickshaw and CNG
HH02	MW	6	Yes	Diarrhoea, fever	Boy	Mother	24	2	Married	Secondary	Housewife	Nuclear	4	Wage loss	Day labour (father)	Father	10 km	Rickshaw and CNG
HH03	MW	11	Yes	Diarrhoea	Girl	Mother	25	2	Married	Primary	Housewife	Nuclear	4	Mother’s sickness	Carpenter (father)	Father	12 km	Rickshaw and CNG
HH05	MW	16	Yes	Diarrhoea	Girl	Mother	33	3	Married	Secondary	Housewife	Extended	6	Wage loss	Carpenter (father)	Father	15 km	Rickshaw and CNG

Of the 12 rural children involved, 5 were SWK, 4 were MW, and 3 NW, with 8 having experienced a family disruption. Nineteen of the twenty-two children were reported by family members to have fully recovered by the time we completed our interviews and three children had died.

In an earlier publication, we described treatment-seeking pathways for the undernourished children in the CHAIN cohort, and key influences on treatment-seeking actions and recovery [48]. Across what were often lengthy treatment-seeking pathways, we showed that important influences were hospital advice and media campaigns, social and financial support from family members and others, and cost of treatment.

In this paper we present in detail the gender related influences at facility/health and household/community level that interplay to shape specific elements of treatment-seeking pathways for the wider group of 22 children (ie not only those who were undernourished), including when a child is admitted, who brings the child to hospital and stays with him/her during hospitalisation, and adherence to advice post-discharge.

We first present one household story (Table 4) to show how the hospital/health system and community/household gender influences interplay, and how the post-discharge experience for children is inextricably linked to pre-admission and during admission influences.

**Table 4:** A story of one cohort child admitted in icddr,b Dhaka hospital in Bangladesh aged 6 months with SWK (Household – HH53)

Ali (pseudonym) is seven months old. He lived in a rural area with his father and 20 year old mother until his parents divorced when he was 45 days old. The mother, who has primary level education, moved with Ali back to her own parents’ home in a Dhaka slum, to live with her parents, brother and sister. So people were living in one rented room. The room has a tin roof and walls, which amplifies the high temperatures in summer. Ali’s father refused to keep Ali in the rural home and so Ali’s mum claimed financial support from the child’s father for his costs (food, clothes and treatment, in line with national law). Ali’s father refused to pay. His uncle and aunt were the main income earners in the Dhaka house, but their income from working in garment factories was barely enough to meet the basic daily HH needs of their own parents. So, the additional cost for Ali and his mother became a burden for them. In addition, Ali’s mother needed to pay her layer to fight her case against the child’s father. Although she started to work as a maid to try to earn some money, her own mother (Ali’s grandmother) couldn’t care for Ali and manage all the other household chores. No other relatives lived in the area and the family was never visited by a community health worker (CHW). Regarding Ali’s health and development since birth - the grandmother said that “*the child was not getting enough breast milk by 2 month of age and so was crying after feeding”.* She contacted a nearby drug seller she often used because he was friendly and did not ask for tests before giving medicine. He advised the child is given formula to supplement the breastfeeding. They began this. Over the next few months, Ali suffered from diarrhoea several times, lost weight, got ‘bony’ and at 4 months developed measles. To get low cost traditional treatment Ali was taken back to the rural area to stay with his mother while she visited her rural house. There he was recovering and so was returned to Dhaka, but was still weak and so the maternal grandmother gave him vitamin syrup. He soon developed watery diarrhoea. Ali’s mother was worried that her mother was unable to care for her child and so gave up her job as a maid. She was very concerned that if anything bad happened to Ali his father would blame her and submit an objection to the police or court. The drug seller suggested oral rehydration saline (ORS), but even after administering it the child’s condition continued to deteriorate and he started to vomit. So the drug seller referred the child to a hospital, but Ali’s mother did not have the money to go to that hospital and was afraid to travel there alone. A neighbour advised her to go to icddr,b Dhaka for free treatment. Ali’s uncle assisted her with transport costs and accompanied her and the grandmother to icddr,b, where Ali was admitted. In addition to diarrhoea and vomiting, Ali was diagnosed with SWK. He stayed in hospital for 19 days with either his mother or grandmother always there with him. They were assisted by Ali’s uncle with food. A major concern for the mother during the admission was the child’s father phoning to say, *“if anything happens to my son, I will sue you”*. She took special care of her child in the hospital for early recovery. Hospital staff noticed that when the grandmother was there without the mother, she would sleep a lot, as she herself was unwell. The grandmother was worried about the other household members who needed her to cook for them. In the end the household pressures were so great that Ali’s mother and grandmother discharged him against medical advice. They did receive counselling from hospital health workers before discharge on recommended food, medicine, water, hygiene and sanitation practices, and illness prevention strategies. **After discharge, despite nutrition counselling**, the grandmother felt that breast milk and formula were adequate; and that the child did not need complimentary food. The grandmother also admitted feeling so overwhelmed sometimes that she wished the child would be sent to his father so her daughter could remarry. Although the mother and grandmother worked to follow the hygiene practices and use half boil water advised by health workers, we observed this was difficult to maintain, and within a week of discharge the child was suffering again from cough and fever, and on day 14 developed diarrhoea. Ali’s grandmother attributed this to the mother’s constant colds being transmitted through her breast milk to the child and his constantly wet clothes from urination. She bought medicine from a drug seller which reduced the child’s fever but not his cough. Relatives and neighbours advised against them returning to icddrb in case he died but the mother had faith in them so went following some advice with a cohort study clinician over the phone. He was re-admitted directly into ICU and treated for diarrhoea and pneumonia. He was discharged from hospital 12 days later after he showed signs of recovery. Post-discharge the mother prepared food at home as per hospital instruction for 3 months. She could afford this because the father had been given a court order to provide. But on the 3rd visit of the social science team the child appeared sick and thin - his aunt had suddenly lost her job and the mother was suspected by the grandmother to be spending some of the child’s money from the father on herself. His cold at the time was considered by her ‘not to a big problem as he’d had it since birth so would recover’. Unfortunately, the child died 180 days after his first discharge from icddr,b hospital.	

In subsequent sections, we discuss key findings (summarised in Table 5) regarding gender related influences on adherence to advice and treatment-seeking guidance following hospital discharge among infants and young children in Bangladesh.

**Table 5:** Summary of gender related influences adherence to advice and treatment-seeking guidance following hospital discharge

Central theme	Sub-themes	Key findings on gender influences	Implications for post-discharge treatment-seeking, adherence to advice and outcomes
Gender related influences at the facility/health system level	Fewer female medical practitioners available in healthcare facilities, esp. in rural areas	-Mothers’ (esp young, rural married mothers’) hesitant to interact with male providers due to socio-cultural and religious norms; reinforced by other household and community members - More rarely: reports and experience of sexual harassment of women by male providers	• Delays in visiting medical practitioners for a mothers’ own health (with implication for her ability to care for a child) • Delays in visiting medical practitioners when a child is sick
	Male caregivers preferring not to stay in the paediatric ward with (re)-admitted children	-Childcare considered primarily in the female domain, including during hospital admission, and many young children are breast-feeding. - Mothers expected to be present in wards, men’s presence can make mothers uncomfortable - Mothers therefore receive more advice than their male counterparts and other family members on children’s feeding, hygiene and medication practices	• Mothers need to get home from admission as soon as possible to complete their other domestic and income earning roles • Families discharge their children against medical advice and resist re-admission • Mothers do not necessarily have the time or power in households on their return from hospital to implement or to share their knowledge with other household members
	More female than male CHWs in health facilities and in hones	- Some male family members did not value female CHWs’ advice, and some especially opposed their anti- domestic violence work - Some male partners of CHWs did not want their wives entering others’ homes where men are living (concerned about reputation, safety and affairs)	• Female CHWs are a potentially strong support for mothers post hospital discharge. • Potential support pre and post discharged undermined by social norms and undervaluing of CHWs by some
Gender related influences at the household/community level	Gendered roles and relations	-Women’s work includes domestic roles and, esp. in urban areas, income earning. Men rarely assist with ‘women’s work’ - Some women, esp. rural young women from conservative religious homes, are not allowed to move without male escorts outside the home - Women traditionally feed themselves last in homes, and miss out on food if necessary -Women’s body shape sometimes more appreciated/admired prior to breast-feeding	• Delays in visiting medical practitioners for a mothers’ own health (with implications for her ability to care for a child needing special support post discharge) • Delays in visiting medical practitioners when a child is sick • Mothers stop breastfeeding out of concern for their body shape or because they are concerned about inadequate breast milk
	Women’s access to household resources, and decision-making power	-Women often have low or no access and control over household resources, sometimes even income they have earned themselves -Deliberate intention among some men to maintain leadership at home; ‘disobedient’ women risk social/physical abuse, and divorce -Divorced/separated women can be seen as a burden in their parents’ own home, and face particular challenges accessing funds from children’s fathers	• Default decision-makers during child illness often the child’s father and elder women (especially mother in laws) – may have a preference for shop drugs or healers • Difficult for an especially young woman to go against her husband’s choices re feeding and treating children; fears being blamed for any unintended adverse events
	‘Boy preference’	- Much discussion about boy preference linked to their expected future income-earning roles, provision for their parents, and continuation of the father’s lineage - Some women are more respected for giving birth to a boy, and may face stigma or divorce if they do not give birth to a son. - Birth spacing may be reduced if women are trying to give birth to a boy	• Mothers may be better supported by children’s fathers with regards to pre and post discharge food provision and treatment-seeking for boys compared to girls • Mothers may be under greater to discharge their girl children from hospital against medical advice

### Gender related influences at the facility/health system level

#### Fewer female medical practitioners being available in healthcare facilities, particularly in rural areas

There was widespread recognition across participants (household members, community representatives and health care providers) that there are fewer female than male medical practitioners available in healthcare facilities, particularly in rural areas; a reported gender difference documented in national databases [16]. Interviewees gave a range of reasons for this pattern, including that few female practitioners are willing to be posted to remote or rural health care facilities and that family members (particularly conservative Muslim men) are more hesitant to allow female practitioners compared to male practitioners to live remotely.

After discharge from the hospital, many interviewees were concerned that provider gender had an important influence on mothers’ willingness and ability to seek prompt care and advice for themselves, with important indirect implications for child related health and treatment-seeking. This was perceived to be a particular concern for less educated young mothers, and for mothers married to conservative Muslim men. Reasons given for mothers’ hesitation in seeking prompt advice and care from male providers were cultural and religious taboos against unmarried men and women interacting. More rarely, concerns about sexual harassment of women by male health workers were raised.

Recognising women’s concerns about interacting with male practitioners, and the implications for treatment-seeking for children, two male practitioners reported developing strategies to encourage women to visit them, including making special efforts to build rapport with women and seeking assistance from female colleagues. Although they felt these strategies were successful, it was notable from other interviews that mothers’ reluctance to seek care from male practitioners also came from, or was reinforced by, other members of their household or community. For conservative Muslim families in particular, a woman talking with a male physician about her own or her child’s illness was reported to be a sin.

The concerns of mothers and family members about a male medical practitioner impacted on their treatment-seeking for children in important ways, including in delaying access to formal healthcare, and influencing their adherence to recommended care and treatment post-discharge. In two of the households we followed up for example, young mothers who had sought treatment for their child from a male practitioner were later forced by elder family members (a father in law and mother in law respectively) to discontinue the treatment. In both cases the father of the child was absent (migrant workers), and these relatives advised that the mother seek treatment from a female healer instead. As one of these mothers recounted:

*“I received treatment for my child’s diarrhoea from a male physician against the decision of my mother-in-law. For this reason, my mother-in-law had stopped talking with me. So, I had to discontinue the treatment to make my mother-in-law happy and went instead to a female healer as she suggested. [Going there] did not help the child recover.”*Mother, Rural,HH01

For their part, many men were not comfortable taking their children to male physicians because they felt it was their wife’s role. They felt they did not have as in-depth knowledge as their wives of the illness history, and that physicians expect to get such detailed information from women as the usual primary caregivers of children.

Nonetheless, the gender of the provider was not felt to be important for everybody in relation to children’s health and associated treatment-seeking, particularly in urban Dhaka. Some interviewees mentioned that relatively educated, wealthy and employed women, and women who already have significant interactions with men beyond their family, were not as likely as others to be as concerned. Absence of older in-laws with more traditional views was also considered important. One mother explained:

*“I am used to talking with men in my workplace (garment factory) where both men and women work together. My husband does not mind this as he also works in the same working environment. So I never hesitate to talk with a male health worker about the illness of my child, or even about my own health problems.”* Mother, Urban, HH55

It is also noteworthy that the gender of the medical practitioner appeared to be less of a concern for large urban hospitals where children were re-admitted, compared to the smaller local health facilities in rural areas. The reason for this was unclear. We however think that this could be pegged to the severity of the child’s illness by the time they are being re-admitted into these larger hospitals; as well as patients and parents being less isolated in these facilities compared to when in smaller, especially rural, facilities.

#### Male caregivers preferring not to stay in the paediatric ward with (re)-admitted children

We observed, as expected, during initial admission and re-admissions that children in the paediatric wards were generally cared for by female relatives of various ages, with men visiting their admitted children occasionally to bring money, supplies and to catch up on their children’s progress. One of the main reasons for this pattern is that childcare is primarily considered to be in the female domain while income earning is primarily in the male domain. Children’s fathers were often described as needing to be out earning an income to support their family members, especially given the financial needs and constraints related to the child’s illness and admission. Also, many children were still breast feeding and therefore needed their mothers there with them wherever possible.

Where women were employed or had particularly high work burdens in their homes, they reported that they could not ask their husbands to take over from them in the wards because reportedly (by health workers) nurses and physicians did not want fathers there, and fathers also felt uncomfortable about it as they did not want to be the ‘only men’ there. Part of this discomfort for the health staff and parents was that there is little privacy in the wards, and it is difficult for other women to breastfeed their children if there are men in the wards; linked to general cultural and religious norms in this context around what is appropriate ‘mixing of sexes’.

The above challenges contributed to some employed mothers, and some mothers with many other responsibilities in their homes, to discharge their children against medical advice, failing to follow post-discharge advice and resisting re-admission to hospital. As one urban mother explained:

*“My husband does not feel comfortable in the wards and so prefers not to stay there looking after my child during my working hours. He was available to help at that time due to his unemployment but I had to take care of my child during re-admission [in another hospital] for a few days. This led to me losing wages. Because of the income loss I could not purchase the prescribed medicines. In the end I made the decision to take discharge against medical advice.”* Mother, Urban, HH55

As women are allowed and able to stay in the hospital, they generally have much more interaction with facility health providers than male relatives or other family members. They therefore receive much more advice than their male counterparts on children’s feeding, hygiene and medication practices. Nonetheless, once they are discharged and are back home, it can be difficult for them to implement this knowledge. One reason for this is that these women often have multiple chores to attend to and so may have to hand over some of the child’s care to others in the home (without an accompanying handover of the information obtained from the hospital). At the same time, due to household hierarchies and dynamics, their knowledge and views – even if based on information given in hospitals - may not be as highly regarded as that of men and elder women. As an adolescent mother from an extended urban household explained:

*“I was trained during admission in the icddr,b hospital on providing medicine, food, and properly breastfeeding my child. After discharge, I had to get back to doing household chores. Other family members - particularly my mother and father-in-law - took care of my child most of the time. So I could not provide food and medicine directly to my child. I suggested that they wash their hands before preparing and providing food and medicine to my child, but they did not listen. Sometimes I was scared to ask them to maintain the proper child care practices. Since I am the youngest and least experienced woman in childcare in the house, they didn’t really value my suggestions.”* Mother, Urban, HH59

#### Family members’ concerns linked to the gender of community health workers

In contrast to the gender distribution of medical practitioners, there are far fewer male, as compared to female, CHWs in Bangladesh. According to national guidance, government and NGO linked CHWs can support with the well-being and treatment of young children through providing advice on where to seek care, and counselling on food and hygiene practices, including post-discharge from hospital. Nonetheless, very few of the household members we talked to reported consulting or getting support from CHWs, despite many being eligible according to national and local guidance.

Three CHWs aged above 50 years felt that their gender did not impact on their ability to perform their expected tasks, and in fact assisted them to get easy access to households. However, several other younger CHWs, and particularly those with less formal education, reported that they faced a range of challenges. Firstly, they felt that male family members did not value their advice as much as women did, and so when women were out working and only male household members remained in homes, it was uncomfortable to enter homes with children in need. Young and unmarried female CHWs in particular felt shy about and even feared talking alone with older men in households. Furthermore, male CHWs although fewer, also faced gendered concerns regarding interacting with women alone, particularly in communities that did not know them well. As one male CHWs explained:

*“Previously I faced challenges working with women to ensure child vaccine coverage as I was posted in an unknown community. I had to spend a lot of time talking with male household members to establish good rapport to get easy access to the household and to talk to women about the required health services for their children. Now I am not facing such problems as I transferred to work in my own community where everybody knows me very well since birth. [there are no questions of] Who I am? Whose son?.”* Community health worker, Rural, CHW03

Secondly, many volunteer CHWs of BRAC [Bangladesh Rural Advancement Committee-an NGO] are provided support with women’s empowerment and to prevent domestic violence in addition to the services for child well-being. Their services for preventing domestic violence reportedly led to some female CHWs being prevented by their own husbands to go and visit homes (had security concerns for their wives). It also led to some female CHWs being prevented from visiting households by men, with negative implications for CHW’s ability to support children following hospital discharge. As one CHW reported:

*“Some mothers discussed with us about the domestic violence by their husband and wanted suggestions from us [about what they could do]. The husbands then found out that their wives had received suggestions from me about how to handle illegal behaviour. Later I was not able to access those households to perform my regular duties [including child monitoring]. I knew about [the law] because I received training about violence against women from BRAC.”* Community health worker*,* Urban, CHW25

A related concern for female CHWs of BRAC was that they were sometimes restricted by their own partners in going out of their homes to conduct their CHW roles. As already noted above, this was sometimes related to concerns about their women’s empowerment agenda, or more generally to conservative religious views regarding women interacting with non-family males. Other reasons included female CHWs having a heavy burden of responsibilities in their own homes in addition to their voluntary role as CHW, and husbands being concerned about their wives having extramarital relationships.

Thirdly, use of mobile phones is important in getting in touch with children’s mothers. However mobile phones are sometimes controlled by men in households making it difficult to reach women over the phone to provide advice and support. This was especially the case in households having only one mobile phone and in more conservative Muslim families. Male family members were reportedly concerned that access to a mobile phone by young and middle-aged women increased the possibility of an extramarital relationship.

### Gender related influences at the household/community level

#### Gendered roles and relations

Most interviewees reported a strong gender difference in household roles, with many suggesting that women overall work longer hours than men (largely in unpaid care and farm work and sometimes in paid jobs), especially in poor households with few income earners. In addition to the care of their children, women are primarily responsible for regular household chores, farming activities and sometimes (especially in urban areas) income earning work outside the home. In contrast, men were generally reported to play the main income-earning role, working 4 to 8 h in a day outside of the home, and to spend the remaining hours socializing with others, watching television, sleeping and – rarely - assisting their female partner in caring for their children. Given this broad division of roles and responsibilities, it is typically the child’s father who is responsible for paying for a child’s food and health care, and the child’s mother (with the support of other female relatives) is responsible for ensuring that the care and treatment is given.

Interviews with fathers suggested that it is unusual for fathers to directly care for their children or give treatment and that many feel uneasy and inadequately prepared to do so. Furthermore, given societal norms about appropriate gender roles, it might be frowned upon in the local community if a husband is seen to be undertaking these perceived ‘feminine’ tasks. There was also a suggestion that a father may not have a similar level of love for his child compared to a mother.

Interviewees mentioned that this range of responsibilities for mothers can mean that mothers with children admitted in hospital are keen to return home as quickly as possible, contributing to early discharge. For mothers this heavy workload can also contribute to some post-discharge advice not being followed. Where mothers have to go out to earn an income and lack of support from their male partner, particularly in the urban areas where there are more employment opportunities and higher costs of living such as rent, children’s follow up care post-discharge may also be compromised. Two working mothers commented for example that when their children were ill post-discharge, they had to take them to their workplaces because they did not have a suitable caregiver at home. At their workplace - despite wanting to - they were unable to follow hospital instructions, due to work demands.

Several rural interviewees mentioned that women in conservative Muslim families with husbands who have migrated away for work are not allowed to move alone outside of the home and so cannot access a health centre to get care for their ill children unless accompanied by male relatives. For children who are still breast-feeding and advised by hospital health workers to continue this post-discharge to prevent illness and reduce undernutrition, additional gender-related concerns arise. These include norms around lactating women being asked to serve others in the household before themselves particularly in extended families, leading to their going hungry (with negative implications for milk production). Also, young men with low education levels reportedly worry that if their wives breastfeed their children regularly, their breasts and body shapes will be less attractive (contributing to early cessation of breastfeeding).

#### Women’s access to household resources, and decision-making power

In this study context, traditionally and to date, men and elders have financial control over the household’s income and other resources. This applies even to employed women who sometimes cannot access the income that they have earned, as cultural norms dictate that they should give their income to their husbands (see illustrative quote below). Many interviewees mentioned that women’s lack of access to household resources can prevent them from being able to follow hospital advice post-discharge, and work against them bringing their ill children to a physician as needed. One mother explained her situation:

*“As per instructions from my mother-in-law, I am supposed to give my monthly salary to my husband for household expenditure. I told my husband [once during post-discharge] to take my sick child to a physician, but he did not do anything. Later on, I asked him for money so that I could take the child for treatment, but he still would not give it to me. Instead, he beat me for asking for it! Over time, my child’s illness got worse. Fortunately, I was able in the end to borrow some money from my brother and friends and so could bring my son to the hospital where he was admitted because he was so severely ill*”. Mother, Urban, HH55

Accessing funds from a child’s father can be even more challenging where parents are separated or recently divorced. In another situation of divorce in an urban area, the father brought his child forcefully from the child’s mother to live with his family. The carer of the child (an unmarried aunt) could not provide breast milk to the child, which was felt to have contributed to repeated diarrhoea and weight loss of the child post hospital discharge.

Many interviewees described that a child’s father and other household elders (e.g. grandparents) make decisions about all family matters, in particular those with financial implications. This includes decisions about the food, medicine, and treatment-seeking needs of children, including in the post-discharge period. Reasons included women being considered outsiders to a home (having only come in from another home on marriage), and as the carriers of children rather than their main creators. Women’s agency to make decisions regarding care of their children post-discharge was therefore limited in many extended households. Extended family members also helped mothers in caring for children when they were busy with household chores. This agency and assistance was generally reported to increase with age, education, employment and where women were bringing money into the household from their own parents’ home. This highlights important intersections of gender with other social categories to increase women’s ability to play a role in their children’s care and make related decisions.

Beyond generally having low decision-making power, five mothers with relatively low formal education directly reported that their husbands were unwilling to listen to the advice mothers had received during their child’s admission with regards to the types of food the child should be given and where they should go for treatment. They attributed fathers’ unwillingness to listen to a deliberate intention among fathers to maintain their leadership status in household. They said they were unable to challenge their husbands’ behaviour as perceived disrespect of husbands could result in violence. Four mothers for example reported being obliged to go to healer or drug-seller for their child’s treatment against the mothers’ wishes. Below is an illustrative quote of one such instance:

*“The father of the child brought medicines from the local drug shopkeeper for my child despite having the prescribed medicines by a hospital physician to continue at home after discharge. He has forbidden me from continuing with the hospital medicines because of the child’s delayed recovery and instructed me to start the drug seller's medicines instead. I was obliged to do it, otherwise he will beat me.”* Mother, Urban, HH54

Mothers, particularly those not earning an income or in precarious relationships, also reported being hesitant to seek treatment for their children from medical practitioners against their husbands’ wishes as they would be blamed for any unintended adverse events (i.e. deterioration of illness condition, treatment failure or death), and risk being beaten or divorced. One mother noted that hospital staff had advised against giving street food to her child to prevent illness (i.e. diarrhoea, fever) or against seeking treatment from healers or untrained medical practitioners. However, family members did not listen to the mother post-discharge, which she felt was unfair, given that she is also blamed when the child gets ill:

*“An uncle of the child brought outside food (low quality bakery food) to feed my child. He quarrelled with me when I told him to avoid those food items to prevent the illness of my child, but he still gave the food to the child. But other family members often blame me when the child gets ill which I think is totally unfair.”* Mother, Urban, HH59

#### Greater reluctance among parents to invest money and time in the treatment of daughters over sons

There was a widespread perception among participants (household members, community representatives, and health care providers) that boy children are generally given more food and medicine, and are better breastfed and cared for, than girl children. During the post-hospital discharge period, in a few households where direct choices had to be made, parents selected care for their older boys over younger girls. The main reasons given for these patterns were that boys are expected to go on to provide financial and non-financial support to their parents in later years and maintain the father’s lineage, whereas girls are expected to leave the family after marriage and provide service in their marital homes as described above.

We observed in our interviews a general difference in the handling of boys and girls, and of more respectful interactions and better support for mothers from their husbands in relation to boy children. For example, during one of the visits to the household of a male child living in the rural site, the father asked his wife to provide medicines to their son in time given that the son is ‘our asset, future, and bank’.

Conversely, there appears to be less support to mothers with girls, with one mother explaining that she had become pregnant too early after the delivery of a girl to fulfil her husband’s desire for a baby boy. Another mother reported being depressed because her husband intended to get another wife in the hope of getting a baby boy. Five mothers reported that in their households, fathers would prioritise their elder boys over girls in buying food in times of significant financial hardship.

In terms of meeting post-discharge treatment-costs and completion of treatment courses, several mothers mentioned that there was a preference for boys, or at least special concern for them. As two mothers explain:

*“My child [girl] was re-admitted to hospital with diarrhoea, pneumonia, and fever. The condition of her illness did not improve after staying for a week in the hospital. The child’s grandmother said, ‘the child’s condition is so bad she’ll not survive, so why you are staying in hospital? Instead you should go back home to look after your elder male children for their future’.”* Mother, Rural, HH05

*“This is our much waited for boy having already given birth to two girls. Recently, my son was admitted to the hospital twice for his illness. We had to spend a lot of money to cover his treatment. His father sold his agricultural land in our rural home to cover it, and he took an urgent loan from a local NGO. We sacrificed our own foods, sometimes eating less and missing out on other basic needs.”* Mother, Urban,HH62

A community representative and health worker reported that girls’ conditions are sometimes more severe due to delayed treatment-seeking, and that there are higher death rates among girl children during re-admission and post-discharge as a result. Interestingly, this pattern was not seen in the CHAIN quantitative data across all cohort children in the two study sites, but some nevertheless describe it as a reality:

*“Female children often die in the hospital due to the delay in treatment-seeking and late re-admission. However, this [the death of a girl child] is not a big matter for parents, but the death of a male child is considered a big loss for them”.* Community representative, Rural, CR-KII-19

One community health worker mentioned that her counselling strategy worked well to motivate some parents to seek early treatment for their girl children from a medical practitioner. She reported giving real-life examples of successful women (e.g., the female prime minister of the country) whose success can be attributed at least in part to their parents’ support.

## Discussion

Contextually relevant gender analyses are needed to improve health-related interventions programs and policies that support children’s recovery following discharge from hospital, but few studies have specifically explored this. We drew on interviews with family members of children who had been admitted in two icddr,b hospitals in Bangladesh, and on interviews with health providers, community representatives and CHWs living in the catchment areas of these hospitals, to document gender-related influences on post-hospital discharge adherence to advice and treatment-seeking for acutely ill young children. In Table 6 we summarise the findings using the Morgan et.al. gender framework [11], adapted to distinguish gender influences at the household/community level and at health service/system levels that influence care. Dominant gender norms, ideologies and roles in households, communities and wider society are replicated and reinforced within and across households/communities and health facilities/systems. Furthermore, men and women’s situations, and the way in which these influence treatment-seeking behaviour for children also differ by age, access to formal education and income, marital status, residence and adherence to more conservative religious beliefs. We have illustrated this throughout the findings, including commenting for example that younger mothers sometimes had less influence than older mothers (and grandmothers) in decision-making about feeding and treatment-seeking, and that divorce brought additional social and financial challenges for some mothers. The household story shared illustrates how age of mother, wealth, access to income, place of residence and marital status interplayed to influence her child’s health and health care. There were also particular challenges for mothers from conservative Muslim families in visiting male medical practitioners and CHWs, contributing to delays in accessing care.

**Table 6:** Gender Framework (power relation and drivers of inequality)

Content	In households/communities	In health services/facilities/system
Who has what?	-Husbands, fathers and elders typically have control over resources in households, including income women have earned -Married women often cannot access household resources, influencing their ability to independently follow hospital and health worker advice	-There are fewer female than male medical practitioners (physicians) employed in (especially rural) health facilities, contributing to some discomfort in women accessing those facilities for themselves and their children. - Most CHWs are female; some fathers are dismissive of their advice
Who does what?	-Women/mothers have multiple domestic tasks in the home, including childcare. Some (especially in urban areas) also earn an income. Traditionally young lactating mothers feed themselves last in a household. -Especially young mothers may have to hand over child’s care to others in the household after discharge	-Hospitals and health centres are seen as a women’s domain; men generally do not feel comfortable staying in facilities with their children, even if their wife is earning -Some CHWs are not welcome in homes, particularly where they are seen by men to be undermining their relationship with their wives
How are values defined?	- Men generally seen as heads of households and main breadwinners, and women as child carers - Young women’s views/knowledge on child health etc. generally less valued than men’s and elders; women sometimes blamed for a child’s condition for failing in her ascribed roles - Boys sometimes seen to have greater potential as breadwinners and future support for the family than girls, and so prioritized over girls in access to food, medicine & treatment	-Linked to the way in which values are defined in households/ communities, caring roles in the health professions are possibly seen as more appropriate and feasible than clinical leadership positions - Positions which can be isolated or need other family members to accompany the job holder (like postings to rural facilities) can be considered more appropriate for men than women
Who made the decisions?	-Husbands and household elders typically made decisions on division of labor in household in relation to children, and on food, medicine and treatment seeking decisions	-Husbands and household elders can influence women’s ability to access medical practitioners and CHWs -There may be greater eagerness or pressure on mothers to get out of hospitals and back to homes where boys are left at home, and where girls are admitted
How is power negotiated and changed	-Especially in urban areas, more women are earning an income exposing them to different types of interactions with men and giving some greater decision-making power over treatment-seeking - Women reported that they were rarely able challenge gender based decision-making norms regarding childcare and treatment because doing so is considered unacceptable socially and culturally.	-Some providers introducing initiatives aimed at easing discomfort of women and their husbands in attending facilities -Some women are interacting with CHWs in empowerment programmes aimed at supporting greater equity, and less abuse, in homes

In terms of health systems/services, two important influences on post-hospital discharge adherence to advice and treatment-seeking for young children were the gender of medical practitioners in facilities and of CHWs. Interviewees reported that concerns about interactions between men and women led to mothers - especially younger rural mothers and those living in conservative Muslim homes - being uncomfortable in seeking care from male medical practitioners, contributing to delays in seeking care for their children, including post-hospital discharge. A mixed-method study conducted in 12 rural sub-districts of Bangladesh reported that most healthcare providers (60%) managing under-5 year old sick children are male [16]. Another survey conducted in four rural sub-districts noted that many women are only allowed to meet an unknown male health provider to seek care for their children in a dire emergency [49]. Together with our study, these findings suggest that it is essential to continue to work towards improving gender parity in who is providing health care for women and children in facilities, particularly in rural facilities, and that in clinical encounters those who wish to see a female practitioner, or have one present, can.

Immediate interventions that could support greater equality include interview and selection processes that recognize the need for more female providers, family-friendly work environments to encourage women to apply for advertised positions (for example flexible work schedules, job shares, and creches at work), and more gender-sensitive career progression opportunities (including distance-learning and part time training opportunities) [50]. Such initiatives are likely to be essential in many LMICs, given the evidence that women’s increased participation in the workforce can help reduce child mortality [51].

In another Muslim majority country, Pakistan, efforts to introduce more female health workers were undermined by abusive hierarchical management structures, disrespectful interactions – including sexual harassment from male colleagues/supervisors, and inadequate consideration of women’s gender-based cultural constraints [17]; challenges that may also be faced in Bangladesh. Any policy changes need to be informed by specifically tailored research into these issues and potential underlying structural drivers of inequality (returned to below). Based on existing literature and our study findings, we suggest that interventions should include a strong emphasis on communication training and support for all medical practitioners, to understand how gender affects their interactions with community members, and what might be done to overcome challenges encountered. Several male practitioners suggested that strategies such as medical practitioners engaging fathers can be valuable in building relationships and trust, which supports study findings elsewhere [52]. Health workers could also encourage women to bring their male relative to a child’s consultation, and local gate-keepers and religious leaders could be encouraged to ease concerns about men and women interacting in health facilities. These initiatives could be supported through mass media campaigns providing gender transformative health education. Such initiatives [53, 54] can reach health workers and gate-keepers as well as other community members, supporting the re-enforcement of key messages.

More in-depth gender analyses at health facility level are needed to inform locally appropriate gender awareness programs at hospital and primary health care levels. This is important given the recognition that health systems are not gender blind, but rather reflect and reinforce societal gender norms [55].

Partly as a result of the above findings, the role of CHWs is potentially particularly important in supporting households (especially women) with identifying and responding to child illnesses. CHWs can also support women in negotiating with others in the household for advisory and financial support, and against emotional and physical violence. While CHWs being primarily women made them relatively accessible to and appreciated by mothers, their advice and support, and sometimes even their visits, were reportedly dismissed by some husbands and fathers. Husbands of CHWs themselves were also sometimes hesitant about their work and concerned that it might lead to their wives’ inappropriate interaction with men. Such findings have been documented before in rural Bangladesh [56], and a cohort study among BRAC CHWs reported that reasons for attrition among female CHWs included family disapproval of their activities and their conflict with commitments to their own family [57].

Previous studies have shown that support and encouragement by family members and supervisors is an important motivating factor for CHWs [56], assisting them to gain confidence in interacting with men and in negotiating work-related challenges [58]. These findings suggest a gender sensitization program is required not only for health facilities, but also for households that CHWs visit, and for household members of the CHWs themselves. Such initiatives could help build trust and respect in the work of CHWs, and enhance their motivation and ability to perform their roles. ‘Courtyard meetings’ are one potential intervention, where parents and elder family members have an opportunity to discuss the role and responsibilities of female (and male) CHWs in relation to family health. Such initiatives are urgently needed in Bangladesh, given the demonstrated potential for CHWs to reduce child mortality in the country and elsewhere [19, 20, 22, 58].

These findings suggest that the interplay between health systems/service and household/community level gender influence post-hospital discharge adherence to advice and treatment-seeking for young children. At household level, women’s gendered roles and responsibilities were exacerbated in the post-discharge period by the need to continue caring for the recovering child, including for example ensuring appropriate and adequate breastfeeding and other feeding, administering medication and following treatment-seeking advice. Many reported an inability to personally oversee all of these tasks, and having to ask for permission for actions requiring funds or visits to providers outside the home. Our findings reflect those of others who have observed that managing nutrition related-childcare practices is particularly difficult for mothers in busy periods such as rice harvesting months [59] and that traditional attitudes of husbands or mothers-in-law can go against mothers’ treatment-seeking desires and cause delays [60].

In LMICs, women’s lower status is positively associated with child mortality rates, highlighting the need to prioritize women’s rights and autonomy [61]. Underlying norms surrounding treatment-seeking and support in child illness, including post-hospital discharge, are gender-based norms of resource distribution and the general valuing of men’s and elder’s views and opinions over those of women, and especially younger mothers. As has also been noted by others, women’s lack of access to household resources (even their own earned money) and their low decision making power undermines their ability to care for and promptly treat their children when they are ill including adhering to post-hospital discharge advice [24].

Women were rarely reported to openly challenge gendered norms because it would be considered socially and culturally unacceptable and could potentially be met with violence or even divorce. This would be especially likely if a child’s condition deteriorated or he/she passed away. This can be a vicious circle. Where mothers suffer from physical violence, this can negatively impact on child outcomes. A study from India for example based on nationally representative data reported that infant and child mortality was greater among those whose mothers had experienced violence from their male counterpart [62]. Violence against women is an important and pervasive problem globally with one report indicating that 1 in 5 women and girls between the ages of 15 and 49 had experienced physical or sexual violence by an intimate partner within a 12-month period [63].

These household/community level findings, and their interaction with health system influences, suggest that health related interventions must engage men and promote gender equity. Such interventions must not be gender-blind, whereby gender inequities are ignored and sometimes even reinforced. Instead, they should be gender transformative. Furthermore, where child health interventions are women-centred with little consideration or engagement of men, (harmful) gender norms and stereotypes around childcare and who is responsible for child health can be deliberately or unintentionally reinforced [64]. We suggest men must come to be seen, and see themselves, as active agents of change in supporting their female partners (mother of the children) in raising their children, and in following post-discharge medical advice when they are acutely ill [52, 65]. Valuable lessons can be learned from the successes of intervention studies in the country that have begun to do this [66].

More fundamentally, our findings support others [23, 24] in demonstrating that the patriarchal norms that limit women’s access to household resources and participation in making decisions for the health and well being of all acutely ill children, and especially girls, must be challenged. More immediate household and facility level interventions such as courtyard sensitization meetings must also be accompanied by fundamental and structural changes at higher levels nationally and globally. Structural drivers of gender inequity include the socio-economic and political processes that structure hierarchical power relations, stratifying societies based on class, occupational status, level of education, gender, and other social categories. As George et al. emphasise, when implemented over time, policies that tackle structural determinants, for example regarding access to education and employment, can achieve long term population effects and reach wider coverage than those (only) focused on household, community or facility level action [67]. Action on these structural influences is therefore essential to maximise and sustain the effect of any more immediate interventions.

An analysis of how gender intersects with other unequal relations of power based on social categories of class, age, educational and occupational status to shape women’s experiences of childbearing and accessing health care for their children would have enriched our analysis and findings. We suggest future studies prospectively apply an explicit intersectionality analysis to better understand and examine the issues presented in this manuscript. Future studies should also complement the focus of this paper on family and community experiences and the post-discharge period with an examination of how health care institutions are influenced by, and reinforce gendered societal norms (including harmful norms).

## Conclusion

There are multiple gender-related influences on healthcare practices for undernourished children during hospitalization and post-discharge in Bangladesh. A complex web of gender related influences operate at both health systems/services level and at household/community level, with potentially important implications across the treatment-seeking pathway for sick children and their families, including post-discharge adherence to medical advice, treatment-seeking and recovery. More immediate interventions with potential to support positive change include the introduction of training and support processes in homes, communities and facilities that build awareness of how gender impacts on child health and recovery, and about how adverse consequences of gender-based discrimination can be alleviated. Specific initiatives include those focused on communication and those targeting changes in routine practices in facilities (including increasing the number of female health workers) and in homes. It is essential that men are considered and ideally incorporated in these initiatives and that in designing and implementing these more immediate interventions, structural determinants are also taken into account.

Ultimately, actions on structural influences are necessary to maximise and sustain the impact of any more immediate actions and interventions. This includes demonstrating and challenging the patriarchal norms that limit girls’ access to education, and women’s access to employment, resources and their decision-making power at all levels. To achieve equality and empowerment of all women and girls, discriminatory laws and social norms need to be transformed, and there must be stronger female representation at all levels of leadership. Unless women’s rights and overall status vis-à-vis men are transformed, the positive effects of more immediate interventions to support children’s recovery post-discharge will be difficult to scale-up and sustain. Equality and empowerment is essential to achieving healthy lives and promotes well-being for all, and particularly for young children.

## Data Availability

The date sets cannot be shared publicly due to institutional roles and regulations. Data generated during the study will be provided to interested researchers (Recipients) from the corresponding author on reasonable request.

## Abbreviations

LMICs: Low and middle-income countries
CHAIN: Childhood acute illness and nutrition network
CHW: Community health workers
icddr,b: International centre for diarrhoeal disease research, Bangladesh
SWK: Severely wasted or kwashiorkor
MW: Moderately wasted (MW)
NW: Not wasted
HH: Household
NGO: Non-governmental Organizations
KII: Key informant interview
UN: United Nation
SDG: Sustainable development goal

## References

[1] Kerac M, Bunn J, Seal A, Thindwa M, Tomkins A, Sadler K (2009). Probiotics and prebiotics for severe acute malnutrition (PRONUT study): a double-blind efficacy randomised controlled trial in Malawi. Lancet.

[2] Maitland K, Berkley JA, Shebbe M, Peshu N, English M, Newton CRJC (2006). Children with severe malnutrition: can those at highest risk of death be identified with the WHO protocol?. PLoS Med.

[3] Talbert A, Thuo N, Karisa J, Chesaro C, Ohuma E, Ignas J (2012). Diarrhoea complicating severe acute malnutrition in Kenyan children: a prospective descriptive study of risk factors and outcome. PLoS One.

[4] Fergusson P (2009). Tomkins A. HIV prevalence and mortality among children undergoing treatment for severe acute malnutrition in sub-Saharan Africa: a systematic review and meta-analysis. Trans R Soc Trop Med Hyg.

[5] Moïsi JC, Gatakaa H, Berkley JA, Maitland K, Mturi N, Newton CR (2011). Excess child mortality after discharge from hospital in Kilifi, Kenya: a retrospective cohort analysis. Bull World Health Organ.

[6] Wiens MO, Pawluk S, Kissoon N, Kumbakumba E, Ansermino JM, Singer J (2013). Pediatric post-discharge mortality in resource poor countries: a systematic review. PLoS One.

[7] Berkley JA, Ngari M, Thitiri J, Mwalekwa L, Timbwa M, Hamid F (2016). Daily co-trimoxazole prophylaxis to prevent mortality in children with complicated severe acute malnutrition: a multicentre, double-blind, randomised placebo-controlled trial. Lancet Glob Health.

[8] Illness CA (2019). Childhood Acute Illness and Nutrition (CHAIN) Network: a protocol for a multi-site prospective cohort study to identify modifiable risk factors for mortality among acutely ill children in Africa and Asia. BMJ Open.

[9] Chisti MJ, Graham SM, Duke T, Ahmed T, Faruque ASG, Ashraf H (2014). Post-discharge mortality in children with severe malnutrition and pneumonia in Bangladesh. PLoS One.

[10] World Health Organization. What do we mean by “sex” and “gender”? 2014. https://www.legal-tools.org/doc/a33dc3/pdf/. Accessed 18 December, 2019.

[11] Morgan R, George A, Ssali S, Hawkins K, Molyneux S, Theobald S (2016). How to do (or not to do)… gender analysis in health systems research. Health Policy Plan.

[12] George A (2008). Nurses, community health workers, and home carers: gendered human resources compensating for skewed health systems. Glob Public Health.

[13] Molyneux CS, Murira G, Masha J, Snow RW (2002). Intra-household relations and treatment decision-making for childhood illness: a Kenyan case study. J Biosoc Sci.

[14] Tolhurst R, Amekudzi YP, Nyonator FK, Squire SB, Theobald S (2008). “He will ask why the child gets sick so often”: The gendered dynamics of intra-household bargaining over healthcare for children with fever in the Volta Region of Ghana. Soc Sci Med.

[15] Kabia E, Mbau R, Muraya KW, Morgan R, Molyneux S, Barasa E (2018). How do gender and disability influence the ability of the poor to benefit from pro-poor health financing policies in Kenya? An intersectional analysis. Int J Equity Health.

[16] Billah SM, Saha KK, Khan ANS, Chowdhury AH, Garnett SP, Arifeen SE (2017). Quality of nutrition services in primary health care facilities: Implications for integrating nutrition into the health system in Bangladesh. PLoS One.

[17] Mumtaz Z, Salway S, Waseem M, Umer N (2003). Gender-based barriers to primary health care provision in Pakistan: the experience of female providers. Health Policy Plan.

[18] Wharton-Smith A, Rassi C, Batisso E, Ortu G, King R, Endriyas M (2019). Gender-related factors affecting health seeking for neglected tropical diseases: findings from a qualitative study in Ethiopia. PLoS Negl Trop Dis.

[19] Tripathi A, Kabra S, Sachdev H, Lodha R (2016). Home visits by community health workers to improve identification of serious illness and care seeking in newborns and young infants from low-and middle-income countries. J Perinatol.

[20] Gogia S, Sachdev HS (2010). Home visits by community health workers to prevent neonatal deaths in developing countries: a systematic review. Bull World Health Organ.

[21] Sarma H, Jabeen I, Luies SK, Uddin MF, Ahmed T, Bossert TJ, Banwell C (2020). Performance of volunteer community health workers in implementing home-fortification interventions in Bangladesh: A qualitative investigation. PLoS One..

[22] Baqui AH, Ahmed S, Arifeen SE, Darmstadt GL, Rosecrans AM, Mannan I (2009). Effect of timing of first postnatal care home visit on neonatal mortality in Bangladesh: a observational cohort study. BMJ.

[23] Burtscher D, Burza S (2015). Health-seeking behaviour and community perceptions of childhood undernutrition and a community management of acute malnutrition (CMAM) programme in rural Bihar, India: a qualitative study. Public Health Nutr.

[24] Chisti MJ, Shahid ASMSB, Shahunja K, Das SK, Faruque ASG, Ahmed T (2018). Mortality rates from severe acute malnutrition requiring hospitalisation is higher in the children of working mothers in Bangladesh. Acta Paediatr.

[25] Muraya KW, Jones C, Berkley JA, Molyneux S (2016). Perceptions of childhood undernutrition among rural households on the Kenyan coast–a qualitative study. BMC Public Health.

[26] Hirani SAA, Karmaliani R (2013). The experiences of urban, professional women when combining breastfeeding with paid employment in Karachi, Pakistan: a qualitative study. Woman Birth.

[27] Basu AM, Basu K. Women's economic roles and child survival: the case of India. Health Transit Rev. 1991:83–103. 10148805

[28] Lata LN, Walters P, Roitman S. The politics of gendered space: social norms and purdah affecting female informal work in Dhaka, Bangladesh. Gender Work Organ 2020;1–19. DOI: 10.1111/gwao.12562

[29] Naheed A, Breiman RF, Islam MS, Saha SK, Naved RT (2019). Disparities by sex in care-seeking behaviors and treatment outcomes for pneumonia among children admitted to hospitals in Bangladesh. PLoS One.

[30] Chen LC, Huq E, D'Souza S. Sex bias in the family allocation of food and health care in rural Bangladesh. Popul Dev Rev. 1981:55–70.

[31] Hossain MM, Glass RI (1988). Parental son preference in seeking medical care for children less than five years of age in a rural community in Bangladesh. Am J Public Health.

[32] Pandey A, Sengupta PG, Mondal SK, Gupta DN, Manna B, Ghosh S, et al. Gender differences in healthcare-seeking during common illnesses in a rural community of West Bengal, Indian. J Health Popul Nutr. 2002;20(4):306–11. 12659410

[33] Ahmed SM, Adams AM, Chowdhury M, Bhuiya A (2000). Gender, socioeconomic development and health-seeking behaviour in Bangladesh. Soc Sci Med.

[34] Salway S, Jesmin S, Rahman S (2005). Women's employment in urban Bangladesh: A challenge to gender identity?. Dev Change.

[35] Al-Amin M, Mathbor GM (2019). Agency, empowerment and intra-household gender relations in Bangladesh: Does market-oriented microcredit contribute?. Asian J Womens Stud.

[36] Bhuiya A, Mushtaque A, Chowdhury R, Momen M, Khatun M (2005). Marital disruption: determinants and consequences on the lives of women in a rural area of Bangladesh. J Health Popul Nutr.

[37] Das SK, Faruque ASG, Chisti MJ, Malek MA, Salam MA, Sack DA (2012). Changing trend of persistent diarrhoea in young children over two decades: observations from a large diarrhoeal disease hospital in Bangladesh. Acta Paediatr.

[38] Huda FA, Ahmed A, Dasgupta SK, Jahan M, Ferdous J, Koblinsky M (2012). Profile of maternal and foetal complications during labour and delivery among women giving birth in hospitals in Matlab and Chandpur, Bangladesh. J Health Popul Nutr.

[39] Fakir AM, Khan MWR (2015). Determinants of malnutrition among urban slum children in Bangladesh. Health Econ Rev.

[40] Bangladesh Bureau of Statistics (BBS). Population and housing census-2011. National volume −3: urban area report. Statistics and information division, Ministry of Planning. Government of the people’s republic of Bangladesh. 2014, ISBN-978-984-519-036-7. (Accessed date: 20 Novermber 2020; http://www.bbs.gov.bd/site/page/47856ad0-7e1c-4aab-bd78-892733bc06eb/Population-and-Housing-)

[41] Ferdous F, Das SK, Ahmed S, Farzana FD, Malek MA, Das J (2014). Diarrhoea in slum children: observation from a large diarrhoeal disease hospital in Dhaka, Bangladesh. Trop Med Int Health..

[42] icddr,b. Health and Demographic Surveillance System – Matlab, v. 48. Household Socio-Economic Census 2014. Dhaka: icddr,b, 2016[icddr,b, HDSS, 2014] (Accessed date 20 November, 2020; https://www.researchgate.net/publication/303736934_Matlab_Household_Socio-Economic_Census_2014_Scientific_Report_No_132_-_March_2016)

[43] Arifeen SEL, Baqui AH, Victora CG, Black RE, Bryce J, Hoque DME, Chowdhury EK, Begum N, Akter T, Siddik A (2008). Sex and socioeconomic differentials in child health in rural Bangladesh: findings from a baseline survey for evaluating integrated management of childhood illness. J Health Popul Nutr.

[44] Joshi S, Schultz TP (2013). Family planning and women’s and children’s health: Long-term consequences of an outreach program in Matlab, Bangladesh. Demography.

[45] Ayres L, Kavanaugh K, Knafl KA (2003). Within-case and across-case approaches to qualitative data analysis. Qual Health Res.

[46] Green J, Thorogood N. Qualitative methods for health research. London: SAGE Publications Ltd; 2018.

[47] Zakayo SM, Njeru RW, Sanga G, Mary N. Kimani, Charo A, et al. Vulnerability and agency across treatment-seeking journeys for acutely ill children: how family members navigate complex healthcare before, during and after hospitalisation in a rural Kenyan setting. Int J Equity Health 19, 136 (2020). https://doi.org/10.1186/s12939-020-01252-x 10.1186/s12939-020-01252-x PMC7418306 32778121

[48] Uddin MF, Molyneux S, Muraya K, Jemutai J, Berkley JA, Walson JL, et al. Treatment-seeking and recovery among young undernourished children post-hospital discharge in Bangladesh: a qualitative study. 2020. (In press). 10.1371/journal.pone.0274996 PMC9506605 36149880

[49] Balk D (1994). Individual and community aspects of women's status and fertility in rural Bangladesh. Popul Stud.

[50] Muraya KW, Govender V, Mbachu C, Uguru NP, Molyneux S (2019). Gender is not even a side issue… it’sa non-issue’: career trajectories and experiences from the perspective of male and female healthcare managers in Kenya. Health Policy Plan.

[51] Iqbal N, Gkiouleka A, Milner A, Montag D, Gallo V (2018). Girls’ hidden penalty: analysis of gender inequality in child mortality with data from 195 countries. BMJ Glob Health.

[52] Sarin E, Maria A (2019). Acceptability of a family-centered newborn care model among providers and receivers of care in a Public Health Setting: a qualitative study from India. BMC Health Serv Res.

[53] Parveen S (2007). Gender awareness of rural women in Bangladesh. J In Womens Stud..

[54] Hossain MM, Mani KKC, Islam MR (2015). Prevalence and determinants of the gender differentials risk factors of child deaths in Bangladesh: evidence from the Bangladesh demographic and health survey, 2011. PLoS Negl Trop Dis.

[55] Percival V, Dusabe-Richards E, Wurie H, Namakula J, Ssali S, Theobald S (2018). Are health systems interventions gender blind? examining health system reconstruction in conflict affected states. Glob Health.

[56] Rahman SM, Ali NA, Jennings L, Seraji MHR, Mannan I, Shah R (2010). Factors affecting recruitment and retention of community health workers in a newborn care intervention in Bangladesh. Hum Resour Health.

[57] Alam K, Oliveras E (2014). Retention of female volunteer community health workers in Dhaka urban slums: a prospective cohort study. Hum Resour Health.

[58] Steege R, Taegtmeyer M, McCollum R, Hawkins K, Ormel H, Kok M (2018). How do gender relations affect the working lives of close to community health service providers? Empirical research, a review and conceptual framework. Soc Sci Med..

[59] Levinson M, Halpern O, Mahmud Z, Chowdhury SA, Levinson FJ. Nutrition-Related Caring Practices and Women's Time Constraints: A Study in Rural Bangladesh. Working Papers in Food Policy and Nutrition 18.Friedman School of Nutrition Science and Policy, 2002. 2002.

[60] Stewart MK, Parker B, Chakraborty J, Begum H (1993). Acute respiratory infections (ARI) in rural Bangladesh: perceptions and practices. Med Anthropol.

[61] Brinda EM, Rajkumar AP, Enemark U (2015). Association between gender inequality index and child mortality rates: a cross-national study of 138 countries. BMC Public Health.

[62] Silverman JG, Decker MR, Cheng DM, Wirth K, Saggurti N, McCauley HL (2011). Gender-based disparities in infant and child mortality based on maternal exposure to spousal violence: the heavy burden borne by Indian girls. Arch Pediatr Adolesc Med..

[63] Manandhar M, Hawkes S, Buse K, Nosrati E, Magar V (2018). Gender, health and the 2030 agenda for sustainable development. Bull World Health Organ.

[64] Younes L, Houweling TAJ, Azad K, Kuddus A, Shaha S, Haq B, Nahar T, Hossen M, Beard J, Copas A, Prost A, Costello A, Fottrell E (2015). The effect of participatory women's groups on infant feeding and child health knowledge, behaviour and outcomes in rural Bangladesh: a controlled before-and-after study. J Epidemiol Community Health.

[65] Owais A, Schwartz B, Kleinbaum DG, Suchdev PS, Faruque ASG, Das SK, Rahman S (2017). A nutrition education program in rural Bangladesh was associated with improved feeding practices but not with child growth. J Nutr.

[66] Nguyen PH, Kim SS, Sanghvi T , Mahmud Z, Tran LM , Shabnam S, et al. Integrating nutrition interventions into an existing maternal, neonatal, and child health program increased maternal dietary diversity, micronutrient intake, and exclusive breastfeeding practices in Bangladesh: results of a cluster-randomized program evaluation. J Nutr 2017;147(12):2326–2337. doi: 10.3945/jn.117.257303. Epub 2017 Oct 11. 10.3945/jn.117.257303 PMC5697969 29021370

[67] George AS, Amin A, de Abreu Lopes CM, Ravindran TKS. Structural determinants of gender inequality: why they matter for adolescent girls’ sexual and reproductive health. BMJ. 2020;368.

